# A review of experimental design in forensic taphonomy: moving towards forensic realism

**DOI:** 10.1080/20961790.2020.1792631

**Published:** 2020-08-13

**Authors:** Kelly L. Miles, Devin A. Finaughty, Victoria E. Gibbon

**Affiliations:** a Department of Biology, University of New Brunswick, Fredericton, NB, Canada; b School of Anthropology and Conservation, University of Kent, Canterbury, UK; c Department of Human Biology, University of Cape Town, Cape Town, WC, South Africa

**Keywords:** Forensic sciences, forensic taphonomy, decomposition, *Daubert* standard, exclusion cages, clothed pigs, carrion biomass, scavenging

## Abstract

Forensic taphonomy as a discipline requires standardization to satisfy *Daubert* criteria for scientific data to be admissible in court. In response, there has been a shift towards quantification of methodology and estimating the postmortem interval. Despite these advances, there are still biases and limitations within the discipline not explicitly addressed in the early stages of experimental design nor in final published works. In this article, unresolved debates with respect to the conductance and reporting of forensic taphonomic research are reviewed, beginning with the nature of experimental cadavers, human or animal analogues and their body size, and second, the forensic realism of experimental setups, specifically with respect to caging, clothing and number of carcases. Pigs, albeit imperfect, are a good model to gain a general idea of the trends that may be seen in humans in subsequent validation studies in facilities where human donors are available. To date, there is no consensus among taphonomists on the extent of the effect that body mass has on decomposition progression. More research is required with both human cadavers and non-human analogues that builds on our current knowledge of forensic taphonomy to answer these nagging questions. This will enable the discipline to make the reliable assumption that pigs and donor decomposition data can be applied to homicide cases. A suite of experimental design aspects is suggested to ensure systematic and standardized data collection across different biogeoclimatic circumstances to identify and quantify the effects of potential confounding variables. Such studies in multiple, varied biogeographic circumstances with standardized protocols, equipment and carrion will facilitate independent global validation of patterns. These factors are reviewed to show the need for adjustments in experimental design to ensure relevance and applicability of data within locally realistic forensic situations. The initiation of a global decomposition data network for forensic taphonomists is recommended.

**Key points**
- Pigs are a valuable, albeit imperfect, proxy for human decomposition studies.
- There are few or conflicting data on effects of carcase size, carrion ecology, exclusion cages and scavengers.
- We recommend single, clothed, uncaged carcases for baseline research to reflect regionally specific forensic casework.

## Introduction

Forensic taphonomy is the study of human decomposition to determine circumstances and time-of-death, which has become a focus of both experimental research and retrospective analysis of forensic cases for the past 40 years [1]. Early attempts to document and report decompositional changes were limited to qualitative observations in soft tissue changes, but these were subjective and introduced observer variability when multiple people described visual signs of putrefaction. Taphonomy was in its infancy, and there emerged a need to standardize the data to reduce inter-observer variability. This became even more important with the *Daubert* standard in trial law [2]. The *Daubert* standard [3] is a legal rule of evidence that governs the acceptance of expert testimony from a witness who is qualified as an expert by knowledge, skill, experience, training or education as long as four criteria are met: the expert’s knowledge will help understand the evidence and facts, the testimony is based on sufficient facts or data, the testimony is the product of reliable principles and methods, and the expert in question has reliably applied the principles and methods to the facts of the case to render an expert opinion. As forensic evidence is being used in more trials, *Daubert* has increased the threshold that experts must meet to provide evidence to the court to eliminate “junk science” having significant legal implications for both prosecutors and defendants. Decomposition evidence is one type of forensic evidence that plays a role in homicide cases, and standardization of decompositional data began to emerge in taphonomic research, in part to satisfy *Daubert* criteria. Henssge and Madea [4] built upon this further by proposing four criteria for any postmortem interval (PMI) estimation method to gain practical relevance: (1) quantitative measurement of study variables, (2) mathematical description of the method, (3) taking into account influencing factors quantitatively and (4) declaring the precision and proof of precision on independent materials. In response to this, forensic taphonomic research has seen a massive shift towards quantification of methodology and the subsequent PMI estimation methods. Despite the advances this shift has facilitated in our understanding of human decomposition, there are still biases and limitations within the discipline not explicitly addressed in the early stages of experimental design nor in final published works, leading to ongoing contestation of numerous aspects of experimental design. In this article, we present two sections that review unresolved debates with respect to the conductance and reporting of forensic taphonomic research: the nature of experimental cadavers, specifically whether they are human or animal analogues, and the size thereof; and second, the forensic realism of experimental setups, specifically where caging, clothing and number of carcases are concerned. These factors need to be more fully understood and addressed before taphonomic results are reported to ensure relevance and applicability of data within locally realistic forensic situations.

## The nature of experimental carcases

### Human versus animal analogues

Following the advent of human taphonomic facilities, donated human bodies swiftly overtook animal analogues as the preferred model for studying decomposition. This is particularly true where the development of models for estimating the PMI in human forensic cases is concerned. The logic is that the results from such studies are more directly applicable to forensic cases than data derived from research on non-human models. This argument remains to be proven and there are several issues with it, most recently summarized by Matuszewski et al. [5]. As a start, there are biographic disparities between the donor population for experimentation and the general population to which results are meant to be extrapolated, namely age, body mass and underlying medical conditions that may impact decomposition cycles. Related to this is the inability to control sample biographics of donors leading to a lack of uniformity in the experimental sample, reducing statistical rigour and inferential power. Uneven supply poses logistical problems for actualistic research, primarily generation of a sufficient sample size and necessitating considerable investment in storage capabilities. Perhaps most conspicuous, however, are the legal and ethical challenges which prohibit taphonomic research using donated human remains in most countries. In locations where it is possible, these facilities are strictly bound to tightly controlled and transparent ethical contracts with the donor or the donor’s family. It is for these reasons there are few research facilities that can accommodate human cadavers, which is a limiting factor to obtaining forensically applicable data. Globally, there are only 10 anthropological research facilities in four countries (USA, Australia, Canada and the Netherlands) that permit use of donated human remains for taphonomic investigation. Several countries have proposed the creation of similar facilities, including the UK and India, but proponents have struggled to overcome existing legal restrictions and public resistance. Most forensic taphonomists are, thus, constrained by an inability to use human cadavers as research specimens and must instead use animal analogues.

Animal analogues in biomedical research have been used for over a century with mammals as the most frequently used subjects, including mice, rats, cats, dogs, rabbits, and primates. Ethical considerations in medical trials are a principal concern, particularly in surgical and drug trials. It is standard protocol for medical researchers to first use a mammalian model and then proceed to human trials following rigorous safety protocols and peer review. The safety of human patients is of paramount importance. However, animal care guidelines for research must also factor in the guiding principles of the three Rs: reduction, refinement and replacement, such that animal experimentation must be justified, ethically sound and not take place when other methodologies exist that can reliably simulate the animal outcomes [6]. The ethics of using animal models as human analogues in forensic research is the focus of a recent article by Mole and Heyns [6]. The authors summarize the ethical questions of using human research subjects if there are suitable animal model species available, and the use of animal models at all if other alternatives are available. Taking a closer literature survey at peer-reviewed forensic research from 2012 to 2016, 35.3% used rat models and 29.3% used pigs. However, a breakdown of forensic research reveals that taphonomic and PMI studies made up 24.2% of all forensic results, which were almost entirely reported using pigs as human proxies.

Where forensic taphonomic research is concerned, pigs (*Sus scrofa domesticus*) have emerged as a preferred experimental subject over other mammalian models for several reasons. Pigs are significantly easier to use as taphonomic subjects because they are already domestically raised in agricultural farms and harvested for meat by the general public, so their use avoids issues of human ethical concerns as well as avoiding running afoul of local legislation that prohibits the use of humans in non-medical research. The question needs to be addressed as to whether pigs and humans are sufficiently biologically similar to give meaningful decomposition data that can be applied across species, as has been assumed for many years. These are discussed below regarding their appropriateness as biological analogues for decomposition studies based on ecological theory. The topics discussed are the anatomical similarities and differences, validation studies between humans and pigs and carcase size.

Humans and pigs have numerous anatomical similarities of interest to researchers. Both species are monogastric omnivores with distinct small and large intestines and the ability to adapt to varying amounts of dietary plants and meat, unlike rabbit obligate herbivores or cat obligate carnivores. These anatomical similarities are not limited to the digestive systems of pigs and humans. Humans and pigs share integumentary similarities in the amount of hair, or lack thereof, as compared to other hairy or fur-covered mammals. In fact, pig skin has so many anatomical similarities to human skin that they are now routinely used in providing skin grafts for burn patients [7]. Literature shows that pigs and humans also have similar immunological systems and responses to infectious diseases [8–10]. Due to these similarities, pigs have been identified as test subjects for human medical research; they have been extensively used as a tissue source for living human medical advances such as xenotransplantation of heart valves for the past 50 years, bone in dentistry repair and pancreatic islet cells for treatment of type 1 diabetes [11–13]. Yet despite the numerous similarities between human and pig anatomy, there are differences that should be considered when using pigs as proxies for human research for forensic taphonomy studies. Pig and human digestive systems do differ in some respects. In contrast to a human’s average intestinal length of 7.5 m, pigs have 23 m of intestine. Humans and pigs also have very different digestive vasculature, lymphoid tissues, coiling patterns within the abdominal cavity and bacterial loads [14]. Since decomposition is highly reflective of autolysis of tissues as well as endogenous bacterial activity from the gut microflora, the potential exists for patterns and rates of decomposition between human and porcine models to differ.

One tissue type that is important during decomposition is adipose tissue. There are similarities between human and porcine deposition of fat tissues in the body for subcutaneous fat around the abdomen, and omental fat around the heart and kidneys [15, 16]. Both humans and pigs have an average body fat of around 20% with variation between individuals likely [15, 17]. Complex triglycerides stored as body fat will eventually break down into glycerol and fatty acid components. However, closer examination of the composition of pig and human fat reveals specific differences at the molecular level. Dietary fats have some influence on human fatty tissues and are mostly triglycerides predominated by unsaturated fatty acids that include oleic, linoleic and laurel acids. Pig fat triglycerides, by comparison, are mostly saturated fatty acids such as stearic acid [18]. While the same groups of fat molecules were present in both species, their relative proportions within the respective adipose tissues are significantly different. Given both similarities and differences between humans and pigs, researchers using pig proxies ideally need to have those data replicated with human cadavers to either support or refute the porcine results to be valid in forensic cases.

### Taphonomic anatomical validation studies between pigs and humans

Despite the existence of only 10 forensic research facilities that can use human cadavers for decomposition research, the data gleaned from these experiments are of significance, particularly those validating porcine results with human subjects. Interestingly, these results have not shown consistent results with pig decomposition paralleling human data. One study examined differences in decomposing adipose tissues between the two species. Pig fat not only decomposed faster than human fat, but after 30 days human fat has a mineral content of mostly potassium and sodium, while decomposed pig fat contained more potassium and magnesium [18].

Muscle tissue, in addition to fat, is another tissue of taphonomic importance in humans. Lab studies on small amounts (1.5 g) of skeletal muscle tissue buried in soil were assessed for decomposition differences between humans, cow, lamb and pigs [19]. No single animal analogue’s muscle was a reliable surrogate for human muscle, however, lamb, not pig, muscle produced most similar results to human. In a separate study, forensic entomologists found no difference in the growth and developmental rates of blow flies when reared in the lab whether fed human muscle tissue or minced pork [20]. It should be noted that both experiments were conducted in controlled lab conditions, which may not be realistic of whole carrion in the field.

Whole animal proxies dominate forensic taphonomic literature, due to the complexity of biochemical reactions occurring in a decomposing body as a full entity. But again, these studies show mixed results regarding pig *versus* human decomposition. Forensic entomology has had the greatest success in using pigs as human proxies when sampling and validating the action of forensically important insects. Schoenly and Hall [21] undertook a series of studies in the USA to validate the use of pigs as human analogues for the purposes of increasing scientific rigour for forensic entomology in legal cases. They found that forensically important species of insects colonize human and pig remains similarly with no significant differences found until after skeletonization occurred. They did find differences between size classes of pigs, with greater parallels between humans and pig size of over 50 lbs (∼23 kg). These results were later confirmed by Schoenly et al. [22] when testing four entomological sampling methods with human and pig carcases. They found slight, but not statistically significant, preference of the insects to colonize humans compared to pigs.

A more recent entomological study from Australia used both humans and pigs to compare decomposition rates and odour profiles for training cadaver-detection dogs [23]. They found that pigs decomposed differently and more quickly than humans. They also noted that insect activity on the pigs occurred over the entire carcase resulting in a more consistent mass loss down to bony remains. Insect activity on the human remains, on the other hand, had initial colonization in the orifices of the face and groin. Because of this, as the pigs progressed to more uniformly skeletonized remains, humans displayed mummified front torsos while the back of the torso continued into active decay with more tissue loss. Entomological experimentation not only has the advantage of similarity between pigs and humans with insects present at each cadaver but have indirectly provided information on differences in actual decomposition patterns and rates between the two species as carrion.

In other studies, pigs have been found to be close approximations to human decomposition, but with important differences. Connor et al. [14] noted several disparities between the decomposition of both test subjects. Pigs were found to have a more homogenous decomposition as compared to the high variability in the human donors. One reason put forth for this difference was the heterogeneity in human diets as compared to the more uniform diets provided to the pigs at a rearing facility. A second significant difference between pigs and humans was seen during the bloat stage of decomposition. During bloat, as the name suggests, abdominal gasses accumulate during progression of autolytic cellular enzyme activity and gut microbe metabolism. During bloat, 60% of the pig carcases experienced abdominal rupturing due to the increase in abdominal pressure, while none of the human cadavers’ abdomens ruptured. The authors hypothesize that the most likely cause of this rupture seen in pigs is due to differences between digestive anatomies. As previously noted, pigs have triple or more of the intestinal length compared to humans. When factoring in length with the highly coiled pattern of the intestines within the abdomen, pigs have an exponentially higher surface area for gut microflora to produce postmortem putrefactive gases to levels of pressure sufficient to split muscle and skin.

Independently of Connor et al. [14], the University of Tennessee’s forensic taphonomic research group conducted two concurrent sets of experiments with simultaneous deployment of human, pig and rabbit carrion to not only determine decomposition rates between the three species, but to look at the effects of scavenging on different test subjects [24, 25]. They found that rabbits decomposed fastest in the spring, while pigs decomposed fastest in the summer. Humans were more variable in their decomposition patterns and rates and were more likely to mummify, while pigs and rabbits did not mummify during the experiments. Discriminant analysis of decomposition rates found that rabbits formed one cluster, while pigs and humans formed another cluster. This first aspect to the study concluded that while pigs were more similar to humans than rabbits, pigs were still different enough from human decomposition to merit further study [24].

The second aspect of the study examined scavenging, which is a vastly understudied aspect of forensic taphonomy, as it is well understood that scavenging can be a significant contributor to decomposition rates where more than half of tissue mass loss is a direct result of scavenging activity [25]. Data showed not only differences between humans, pigs and rabbits for scavenger type, but this varied by season as well. In Tennessee, raccoons were the dominant scavenger that preferentially grazed on humans over rabbits and pigs, and this effect was most significant in the winter season when food resources are low. Winter scavenging patterns on humans focussed on the limbs, while scavenger activity on pigs was concentrated on the snout and abdomen. This is theorized to be due to differences in human and pig muscle tissue distribution and skin toughness. Interestingly, wildlife ecology studies show raccoons prefer muscle as a protein source in their diets [26] that was seen with scavenging on human cadavers. During the summer season, only some of the human remains were scavenged, while none of the rabbits or pigs were scavenged at all. However, video and photographic data for raccoon scavenging activity on pig and rabbit carcase show raccoons played with the animal remains by poking pig and rabbit carcases and pulled out clumps of rabbit fur. Only when the human remains had no tissue remaining did the raccoons then select the pig and rabbits as food. This difference in preferential scavenging on human remains over proxy species raises interesting questions that require further investigation.

The resounding conclusion from the studies of Connor et al. [14], Dautartas et al. [24] and Steadman et al. [25] was that the rates and processes of decomposition do, indeed, differ between pigs and humans, with that of humans being more variant. Thus, only humans may be used to directly model human decay. However, this did not diminish the value of animal models in taphonomic research, which, as the authors of these studies explain, lies in their ability to facilitate the establishment of baseline data on decomposition and/or ecological processes where none previously exist and human bodies are not available for use in this type of research [14, 24–28]. These seemingly conclusive settlements to the debate have, however, recently been shown to be flawed. In their recent rebuttal, Matuszewski et al. [5] point out that the Connor et al. [14] and Dautartas et al. [24] studies failed to adequately account for confounding variables, notably the influence of variant carcase size, close inter-carcase distances which may have led to cross-contamination of attendant necrophagous insect communities, and inter-annual effects (specifically in Connor et al. [14]). Furthermore, they highlight that both studies incorrectly applied the TBS scale for quantifying decomposition progression and did so without any supporting measure (e.g. periodic percentage weight loss). Moreover, both studies conducted inappropriate statistical analyses, with Connor et al. [14] selecting inadequate models, not reporting 95% confidence intervals and not allowing pig and human carcases to progress to the same extent of decay, while Dautartas et al. [24] committed an error of temporal pseudo-replication (see Michaud et al. [29] for detailed explanation of pseudo-replication and other common statistical processing errors).

The aforementioned illustrates the current difficulties associated with quantifiably accounting for sources of variance and error as previously emphasized by Henssge and Madea [4], and Matuszewski et al. [5] suggest that it is not possible to recommend a universal analogue for human cadavers in decomposition studies yet. Moreover, they emphasize that abandoning non-human analogues is not currently viable due to the myriad of challenges and restrictions currently limiting use of donated human cadavers for experimental taphonomic research and stress the need for further research to address these shortcomings.

Are pigs a perfect analogue in forensic taphonomy? Perhaps not. There are many unknowns with respect to the differences between what is evidenced by actual forensic cases as compared to donor cadavers or pig analogues. However, we do not believe we should throw out the baby with the bathwater, so to speak, and stop using pigs as human proxies. Preliminary research on pigs demonstrably provides a good way of getting a general idea of the trends that might be seen in humans in subsequent validation studies in facilities where human donors are available.

### Carcase size

The use of myriad species as animal models gives rise to another pressing question: does the size of the carcase(es) used influence the rate and/or pattern of decay? This is not a new problem, but as Matuszewski et al.’s [5] analysis highlights, is as-yet unresolved. The first research objectively investigating this question, albeit for the purposes of assessing possible differences in carrion insect communities, was that of Kneidel [30]. Through comparisons of his own work with previous taphonomic research, he highlighted that carcases greatly disparate in size decompose at different rates and with different patterns. They also appeared to host different invertebrate assemblages. Subsequent work by Hewadikaram and Goff [31] indicated that minor variation in carcase size (e.g. ±10 kg) did not significantly influence the rate of decay or the colonizing invertebrate populations. A trend emerged for using carcases of 23 kg of weight [32, 33] to model human decomposition, but this was soon contested [34]. The reason was highlighted by Kneidel [30] almost 15 years prior: a larger carcase takes longer to decompose than smaller ones. This is due to more biomass and the surface area-to-volume ratio (and, thus, the area available to decomposers for consumption) changes with increased body size. Thus, a 23 kg carcase is not going to decompose at the same rate or with the same pattern as a 60 kg carcase, in any environment. This has been proven in numerous contemporary studies and is an essential consideration when designing and interpreting results of taphonomic research [5, 35–38]. Interestingly, it seems this is irrelevant when there is an absence of insect decomposers [34], which rarely occurs in reality. In the absence of a standard for forensic taphonomic research, Matuszewski et al. [5] recommend that carcases larger than 30 kg should be used. The question will, however, remain unanswered until more variables are quantified and considered to disentangle the confounding effect of carcase mass (i.e. size).

To date there is no consensus among taphonomists on the extent that body mass has on decomposition progression. More research is required with both human cadavers and non-human analogues that builds on our current knowledge of forensic taphonomy to answer these nagging questions so the discipline can reliably make the assumptions that pigs and donor decomposition data can be applied to homicide cases. If not directly applicable, it might be possible to use existing experimental data and adjust it by the use of regionally specific correction factors, thus, increasing the usability of existing data to individual regions. In the worst-case scenario, currently published donor data may be refuted when tested against taphonomic subjects that more closely represent the homicide victims seen in a given locale and new avenues of experimental subjects are investigated. The ultimate goal of forensic taphonomy research is to replicate, using the best available methodologies, what is found in forensic casework to yield accurate and precise data for death investigations. The next section of this article outlines what those current practices are, and whether a fine-tuning of existing protocols can improve our decomposition results.

## Forensic realism

### To cage or not to cage?

Differences in decomposition patterns of humans and pigs are not limited to the endogenous activity of the carcase’s own tissues or the mass/size thereof. The majority of decompositional experiments, whether human or mammal proxy, use exclusion cages. These are typically metal frames of sufficient size to cover the body with wire mesh on five sides to exclude scavengers from accessing the tissues, while at the same time permitting natural access to sunlight, wind, precipitation and small insects. The motivation for this varies and is linked to each study’s objectives. Studies seeking to determine the effects of specific variables on the decay process need to control for as many variables as possible, and excluding scavenging is the easiest way to control for it [39–52]. Scavenging is not a uniform occurrence in decomposition scenarios, so some researchers exclude it when establishing baseline data. In such studies, it is often not possible to catalogue all variables within the decomposition ecosystem and determine their interactions. It may be prudent to control for scavenging as a confounder of findings for the preliminary research. The results of such studies cannot, however, be extrapolated to forensic scenarios unless it can be proven that the remains in question were not scavenged. This is often difficult to do, especially where small scavengers are involved, and their artefacts are not obvious. The same applies to remains that are skeletonized and there is no evidence of scavenging on the bone (even if there may have been on the soft tissue).

However, recent research is suggesting that natural scavenging by a variety of animals is considerably underappreciated as an occurrence in decomposition scenarios and is a significant factor accelerating the rates of carrion decomposition [26, 53–58]. As such, the question needs to be asked whether decomposition experiments that use exclusion cages do, in fact, relate to natural decomposition found in forensic cases that are subject to scavenging activity. This matter was not expressly addressed in the literature until Spies et al. [56] published a pilot study demonstrating that scavenging by the Cape grey mongoose (*Galerella pulverulenta*)—a small mammal not previously considered a significant scavenger—could accelerate the rate of decomposition of uncaged carcases by sixfold compared to a caged carcase in the same habitat. The Steadman et al. [25] study, for the first time, provided solid evidence that PMI estimates are significantly impacted when using previously established models where human cadavers are concerned—human PMI estimates are overestimated and pig PMI estimates are underestimated when compared to estimates calculated from studies where scavengers were excluded. It should be noted that terrestrial vertebrate scavenging activity is not restricted to raccoons and mongooses; the literature reports such species as coyotes, bears, opossum, turkey vultures, domesticated dogs, red fox and carrion crows, among others, having taphonomic impacts on human and proxy remains [54–63]. This list is expected to grow as human settlements encroach more and more onto natural habitats and animals become more synanthropic.

It is, thus, important to interpret the data generated in studies where scavengers are excluded through a biased lens, with acknowledgement that the rates and/or patterns of decay observed may be altered by small and/or large vertebrate scavenging. Where caging is implemented but not explicitly necessary, it is prudent to permit scavenger access in follow-up studies aimed at building upon baseline data, as several authors have recommended [46, 64–66]. Alternatively, researchers planning future research of the decomposition ecosystem could work the potential effects of scavenging into their research designs. This may be accomplished by adapting data collection techniques to maintain successful data collection in spite of the disturbance effect of scavenging or leaving at least one carcase uncaged to determine if there are any small vertebrate scavengers in the ecosystem that may need to be studied in greater detail. Baseline studies of indigent fauna pre-carrion deposition may also illuminate the potential for scavenging and help researchers plan accordingly. In doing so, greater strides will be made towards the collective goal of much of the forensic taphonomic and forensic entomological literature: an improvement of our understanding of the decomposition ecosystem with a view to applying the results in forensic scenarios to aid with case resolution.

### To clothe or not to clothe?

Clothing can impact the rate of decomposition, depending on the materials, number of layers and the degree of coverage of the clothing worn. For example, we know that natural fabrics decompose more quickly on a body than synthetic materials [67] and natural fabrics are shown to accelerate adipocere formation on bodies in aqueous environments [68]. However, findings on the importance of clothing are inconsistent. Kelly et al. [45] reported that clothed and wrapped pig carcases in South Africa decomposed differently than unclothed and unwrapped carcases, particularly from an entomology perspective. Fabric-clothed pigs were observed to have eggs laid on them at the same time as unclothed pigs, but clothed pigs had larger maggot masses under the clothing likely due to the fabric retaining moisture and offering protection from direct sunlight. Similar results have been reported by Cahoon [69] and Miller [70] in Tennessee, and later by Voss et al. [71] in Australia. Matuszewski et al. [38], however, examined body mass in conjunction with clothing in Poland and how they interact to impact decomposition and reported clothing to be a minor factor impacting soft tissue loss to the advanced decay stage. Card et al. [72] went further, concluding from their research that the presence of clothing had no practical impact on decomposition. Interestingly, there has even been work done on how biochemical aspects of decomposition impact the breakdown of cadaver-associated textiles as an initial step to using crime scene clothing as a possible source of PMI data [73].

Few studies have examined the impact of clothing on scavenging (which, by extension, would affect decomposition). Carson et al. [74] anecdotally found that clothing presented no barrier where bear scavengers were concerned. Conversely, Cahoon [69] makes anecdotal mention of the fact that clothing inhibited animal (raccoon) activity where it covered the body, with Young et al. [58] reporting similar findings with foxes. However, none of these studies had attempted to quantify the effect of clothing as a barrier to scavengers. Spies et al. [57] sought to address this shortcoming by comparing decomposition of clothed and unclothed carcases in the same environment and season, with quantification of scavenger activity benchmarked against carcase mass loss over time. They demonstrated that the progression of decomposition was closely associated with scavenging by Cape grey mongoose and discovered that these scavengers had a clear preference for unclothed carcases. This suggests that studies that seek to describe the effects of scavenging on decomposition but do not involve clothed carcases may be inaccurate. Accordingly, they recommended that previous studies investigating scavenging activity be interpreted with greater caution, and study designs re-evaluated for future research to include clothed remains. The rationale behind this recommendation is reinforced by the fact that most forensic cases recovered are in a clothed state.

### One or many carcases?

One of the recommendations of Spies et al.’s [57] study was that since mongoose exhibited a preference for unclothed over clothed pig carcases, further research on the local scavenger behaviour was required with a single clothed carrion option—more representative of real-world forensic scenarios—to validate this pattern. Further impetus for this research is that carrion ecology investigations have demonstrated that multi-carcase deployments increase carrion biomass above baseline levels, with as-yet unquantified influences on the decomposition ecosystem [75]. Accordingly, a follow-up study by du Toit et al. [76] deployed a single clothed pig carcase in the same habitat and equivalent season to the Spies et al. [57] study, with quantification of scavenger activity and progression of decomposition (as percentage weight loss over time). The results speak for themselves: the single carcase experienced a 400% increase in scavenging activity and hit 75% mass loss in only 83 days, compared to Spies et al.’s [57] multiple clothed carcases that never reached the 75% mass loss milestone, even after 113 days of exposure. A clear change in pattern of scavenging behaviour was noted, with the single carcase experiencing more visits, longer scavenger hours, longer multi-scavenger visit durations and a shorter decomposition cycle. The authors suggested that using a single carcase produced results that were more forensically applicable. However, they recognized the need to repeat the experiment temporally and across varied circumstances to verify the pattern. Moreover, their specific study site lacks baseline data on scavenging occurrence and activity pre-carrion deposition, meaning the probability and/or extent of scavenger habituation following successive years of carrion deposition cannot be reliably determined. Habituation concerns have been recognized in carrion ecology research [77–79] but have been largely ignored in the forensic taphonomic literature. This is an area requiring urgent attention by the forensic taphonomic research community, given the legal need to quantitatively take influencing factors into account [5], the important role scavengers are increasingly recognized to play in forensic death scenarios, and because of the demonstrable effect altering the amount of carrion biomass may have on scavenging behaviour.

## Conclusion

Having examined the available literature on the use of animal analogues—pigs in particular—in place of human cadavers, the size of carcases (human or non-human), whether they are caged or clothed, and how many are deployed in each experimental circumstance, it is clear that there are still gaps in our understanding of how these aspects of experimental design influence research outcomes. What elements of experimental design should be planned when investigating forensic taphonomy with forensic implications given what we know? We propose a suite of systematic baseline studies for different biogeoclimatic circumstances aimed at identifying and quantifying the effects of potential confounding variables. Pigs of at least 30 kg should be used to most closely imitate human tissues for first instance (or baseline) research. An initial baseline observation study of regional fauna (vertebrate and invertebrate) should be undertaken to facilitate understanding of how carrion deposition influences the local ecosystem. Where potential scavengers are identified, the subsequent study design should include scavenger monitoring. A single clothed uncaged carcase should then be deployed. Thereafter, multiple clothed carcases may be deployed to determine if the pattern of decomposition changes.

Conductance of such studies in multiple, varied biogeographic circumstances with standardized protocols, equipment and carrion will facilitate independent validation of patterns. Human cadaver validation studies may then follow to complete the transition of study findings to forensic usability. This means, though, that we need more forensic taphonomy research facilities globally and researchers should be actively lobbying government and the public to create and fund these much-needed areas of regional specialization to increase our pool of data on human decomposition. Once we have these concrete results, we can begin to tease out the seemingly innumerable factors that impact decomposition to create a more precise, accurate and appropriate system of creating PMI estimates based on decomposition data. As the data for these types of comparisons begin to accumulate and comparisons made between similar geographical areas, it would be advantageous to initiate a global decomposition data network for researchers to share results, collaborate and gain initial information on decompositional patterns that can be modified and refined to have meaningful localized applications on a wide range of individuals, causes of death, interment types and recovery locations and conditions.
